# Supplementary material for: Augmented two-stage estimation for treatment switching in oncology trials: Leveraging external data for improved precision
Source: Stat Methods Med Res. 2025 Sep 30;34(12):2249–69. doi: 10.1177/09622802251374838 (PMC12669390; doi:10.1177/09622802251374838)
Supplement: sj-docx-1-smm-10.1177_09622802251374838 - Supplemental material for Augmented two-stage estimation for treatment switching in oncology trials: Leveraging external data for improved precision [file sj-docx-1-smm-10.1177_09622802251374838.docx]

**R code for simulating a subject for the simulation study.**

###################################################################################

sim_function <- function(trt = 0,

switching = 1,

high_switching = 0,

enddate = 546,

pmix = 0.5,

lambda1 = 12.5,

lambda2 = 10,

gamma1 = 2,

gamma2 = 3,

delta1 = 2,

delta2 = 1,

delta3 = -0.3,

omega = 1.1,

pr_of_poorprog = 0.5,

bias = FALSE,

unmeasured_confounder = FALSE){

# The probability of switching was set at 0.8 for patients in the poor prognosis group,

# and at ----0.3---- (slightly different than the 0.2 used by Latimer)

# for patients in the good prognosis group in scenarios with a moderate switching

# proportion, and at 0.9 and 0.6 for poor and good prognosis patients respectively

# in scenarios with a high switching proportion.

# Probability of poor prognosis 0.5

badprog <- sample(c(0,1), prob=c(1-pr_of_poorprog, pr_of_poorprog))[1]

# Probability of confounder 0.5

confounder <- sample(c(0,1), prob=c(0.5-bias, 0.5+bias))[1]

if(!unmeasured_confounder) {

if(switching==0){

prob_of_switching <- 0

}

if(switching==1){

if(!high_switching){

prob_of_switching <- 0.8*badprog + 0.3*(1-badprog) }

if(high_switching){

prob_of_switching <- 0.9*badprog + 0.6*(1-badprog) }

}

}

if(unmeasured_confounder) {

if(switching==0){

prob_of_switching <- 0

}

if(switching==1){

if(!high_switching){

prob_of_switching <- 0.8*badprog + 0.3*(1-badprog) - 0.2*confounder}

if(high_switching){

prob_of_switching <- 0.9*badprog + 0.6*(1-badprog) - 0.2*confounder}

}

}

# Disease progression times were simulated to equal OS times multiplied

# by a random draw from a Beta(5,10) distribution, so that, on average,

# the duration of an individual’s PFS is equal to about one third of their OS.

TTP_multiplier <- (rbeta(1,5,10))

f <- function(t) (1-(pmix*exp(-lambda1*t^(gamma1)) +

(1-pmix)*exp(-lambda2*t^(gamma2)))^exp(delta1*trt +

delta2*badprog + delta3*confounder))

f.inv <- inverse(f,lower=0,upper=1000000)

u_sample <- runif(1, 0, 1)

OS <- (c(unlist(lapply(u_sample, f.inv))))

PFS_exact <- OS * TTP_multiplier

PFS_visit <- min( c(OS,ceiling(PFS_exact/12)*12) )

PPS_visit <- OS-PFS_visit

PPS_visit_true <- PPS_visit

# switch?

switch_indicator <- 0

if(trt==0){

switch_indicator <- sample(c(0,1), 1, prob= c(1-prob_of_switching, prob_of_switching))

}

PPS_visit_switch <- PPS_visit

if(switch_indicator){

PPS_visit_switch <- PPS_visit*omega

}

OSswitch <- PFS_visit + PPS_visit_switch

OSswitchstatus <- as.numeric(OSswitch < enddate)

OSswitch <- min(c(OSswitch, enddate))

OSstatus <- as.numeric(OS < enddate)

OS <- min(c(OS, enddate))

TTPexactstatus <- as.numeric(PFS_exact < enddate)

TTPexact <- min(c(PFS_exact, enddate))

TTPvisitstatus <- as.numeric(PFS_visit < enddate)

TTPvisit <- min(c(PFS_visit, enddate))

## PPS is the PPS with the impact of switching

PPSstatus <- 1

PPS <- OSswitch - TTPvisit

if(TTPvisitstatus==0){

PPSstatus <- 0

PPS <- 0

}

if(OSswitchstatus==0){

PPSstatus <- 0

PPS <- OSswitch - TTPvisit

}

return(list(

OS = unname(OS),

OSstatus = unname(OSstatus),

PPS = unname(PPS),

PPSstatus = unname(PPSstatus),

OSswitch = unname(OSswitch),

OSswitchstatus = unname(OSswitchstatus),

TTPexact = unname(TTPexact),

TTPexactstatus = unname(TTPexactstatus),

TTP = unname(TTPvisit),

TTPstatus = unname(TTPvisitstatus),

SWITCH= unname(switch_indicator),

x1 = unname(badprog),

censor_time = enddate))

}
